# Pilot test of an accrual Common Metric for the NIH Clinical and Translational Science Awards (CTSA) Consortium: Metric usefulness

**DOI:** 10.1017/cts.2020.544

**Published:** 2020-09-22

**Authors:** Laura E. Peterson, Denise H. Daudelin, Lisa C. Welch, Anshu Parajulee, Harry P. Selker

**Affiliations:** 1 Tufts Clinical and Translational Science Institute, Tufts University, Boston, MA, USA; 2 Institute for Clinical Research and Health Policy Studies, Tufts Medical Center, Boston, MA, USA

**Keywords:** Performance improvement, common metrics, translational science, study accrual, CTSA

## Abstract

The Common Metrics Initiative aims to develop and field metrics to improve research processes within the national Clinical and Translational Science Award (CTSA) Consortium. A Median Accrual Ratio (MAR) common metric was developed to assess the results of efforts to increase subject accrual into a set of clinical trials within the expected time period. A pilot test of the MAR was undertaken at Tufts Clinical and Translational Science Institute (CTSI) with eight CTSA Consortium hubs. Post-pilot interviews were conducted with 9 CTSA Principal Investigators (PIs) and 23 pilot team members. Over three-quarters (78%) of respondents reported that the MAR could be useful for performance improvement, but also described limitations or concerns. The most commonly cited barrier to MAR use for performance improvement was difficulty in interpreting the single value that is produced. Most respondents were interested in using the MAR to assess recruitment at an individual trial level. Majority of respondents (63%) had mixed opinions about aggregating metric results across the CTSA Consortium for comparison or benchmarking. Collecting data about additional contextual factors, and comparing accrual between subgroups, were cited as potentially helping address concerns about aggregation. Significant challenges remain in ensuring that the MAR can be sufficiently useful for collaborative process improvement. We offer recommendations to potentially improve metric usefulness.

## Introduction

Accrual of participants is critical to the success of clinical research studies, and thus measuring accrual is essential to optimize clinical trial and research organization performance. Studies without sufficient enrollment are unable to evaluate proposed scientific hypotheses, and thus are not a cost-effective use of administrative and clinical resources [1]. The pharmaceutical and biotechnology industries collect and use metrics for trial site selection and ongoing monitoring of trial performance. Such activity addresses the quality of study accrual and conduct across sites for a specific trial, but has limited ability to understand or improve the performance of the larger research organization or system.

There is a growing appreciation that process improvement holds promise for improving research quality and efficiency across the translational continuum [2], including for study accrual. The Common Metrics Initiative aims to develop and field metrics that are used to improve research processes within the National Clinical and Translational Science Award (CTSA) Consortium. After development and pilot testing, each “common metric” is implemented across the CTSA Consortium hubs [3], with the explicit goal of being used for improvement.

The Median Accrual Ratio (MAR) common metric aims to estimate the accrual ratio for a set of clinical trials in order to enhance the hubs’ and the CTSA Consortium’s ability to assess the results of interventions aimed at increasing planned subject accrual into trials within the expected time period [4]. After metric development, a 4-month pilot test was undertaken by the Common Metrics Implementation Team at Tufts Clinical and Translational Science Institute (CTSI). In this paper, we describe pilot study results regarding the usefulness of the metric result for conducting performance improvement for accrual at CTSA Consortium hubs and nationally. A companion paper provides the pilot test methods, and results regarding the feasibility of collecting metric data and the quality of the resulting data.

## Methods

### The Accrual Metric and Pilot Test

**Fig. 1.**
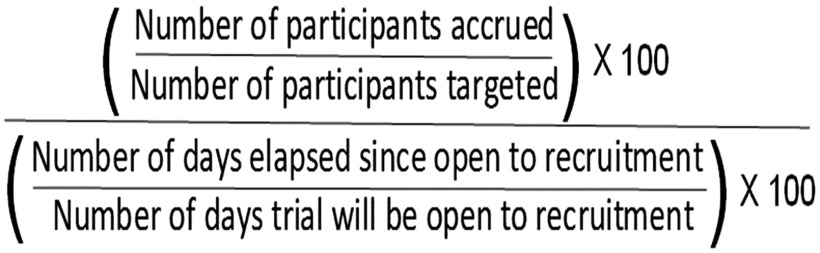
Median Accrual Ratio.

The design of the metric and pilot test procedures are described elsewhere [5]. Briefly, the MAR is the median across a set of clinical trials of the following within-trial ratio (see Fig. 1):

An Operational Guideline specified that the metric be reported annually using data from the prior calendar year, and hubs could elect to report on all, or a subset, of their eligible clinical trials. To test the measure, teams from eight CTSA Consortium hubs participated in a 4-month pilot test including training on the accrual metric Operational Guideline, the performance improvement framework used by the Common Metrics Initiative, and the software program that hubs then used to document the metric result and a performance improvement plan. The Implementation Team also conducted every other week webinars with each of the two groups of hub teams (those from institutions with and without a Clinical Trial Management System [CTMS]). The pilot test was not considered human subjects research by the Tufts Health Sciences Institutional Review Board.

### Assessing Metric Usefulness

To determine the usefulness of the accrual metric, we assessed the extent to which hubs found the metric results understandable, and the metric useful for both performance improvement and public reporting. Interviewees were asked their opinion about the usefulness of the MAR for conducting performance improvement for accrual at four levels:

- Their CTSI
- Their hub’s primary institution
- The level of an individual trial (for an investigator or study team)
- To the CTSA Program Consortium for performance improvement

### Data Collection and Analysis

Data collection included notes taken during webinars and telephonic interviews. Implementation Team members took notes on each of the pilot test webinars, capturing hub questions, barriers, and facilitators as the hub teams attempted to use accrual metric data to initiate performance improvement activities.

After the series of webinars ended, two Implementation Team members conducted semi-structured telephonic interviews with the Principal Investigator (PI) and accrual metric team members from each pilot hub (a total of 9 PIs and 23 team members). Interview topics included actual or potential usefulness of the metric for improving accrual, benefits of participating in the metric pilot, and the feasibility of a comprehensive collection of the metric in the future. Team sizes varied, and interviews were conducted with between one and three team members due to scheduling constraints and participant preferences.

All interviews were recorded with respondent permission. Interviews were transcribed verbatim, reviewed for accuracy, and uploaded into ATLAS.ti 8 software for coding and analysis. Two qualitative analysts used an iterative consensus-based process to develop the codebook. Each analyst independently coded three interview transcripts from respondents at different hubs to identify emergent concepts. They met to compare codes, and after creating an initial codebook, applied it to three other transcripts. Any new concepts in the second set of transcripts were incorporated into the codebook. The two interviewers then reviewed the codebook and definitions for face validity. One analyst applied the final codebook to all transcripts, meeting with the second analyst during the process to resolve any uncertainties. When the codebook was modified during coding, all previously coded transcripts were reviewed to ensure coding consistency across all transcripts.

The analyst who coded transcripts conducted a thematic content analysis for each of the three topics: metric data collection and analysis, using metric results for performance improvement, and respondent suggestions. Themes and sub-themes were identified by reviewing all quotations for codes that occurred most frequently across respondents and hubs [6]. For certain sub-topics, a comparative analysis by respondent role was conducted to determine whether there was a difference in perspectives between PIs and team members [7]. Comparative analyses included frequency counts by role and assessment of differences in content.

## Results

### General Views on Performance Improvement for Clinical Trial Accrual

All interview respondents believed in the importance of measuring and improving clinical trial accrual outcomes at their hub. A respondent explained that improving accrual is essential because failed trials are a “global waste of resources,” including at CTSA institutions that invest funds to help PIs develop trials. It was noted that a wide variety of groups, including CTSAs, home institutions, departments, the NIH’s National Center for Advancing Translational Science (NCATS, which funds CTSAs), and funding organizations want to improve accrual.

We know that PIs and people that manage studies are always concerned about meeting recruitment goals; and anybody that’s in the business knows that it’s an issue.

Some respondents mentioned that the pilot worked synergistically with other efforts to track or improve accrual. These included campaigns to implement an institution-wide CTMS, requests for recruitment updates from study sponsors, and efforts by cancer centers to attain National Cancer Institute (NCI) Cancer Center designation.

### Improvement Plans

As part of the pilot, all hubs developed a performance improvement plan; most described accrual data collection feasibility and quality issues, as well as current accrual barriers and improvement strategies. All but one hub developed its plan in parallel with rather than after data collection. Many respondents reported their discussions during the pilot were much more useful for performance improvement than the actual metric value.

It wasn’t as if we needed the metric result to convince people where we were. They’re pretty much convinced that we have a problem with accrual anyway.

When we did these presentations and talked to people, we really talked to them about the [performance improvement] process and not so much the number [metric value].

### Data Issues Affecting Performance Improvement

Key data collection feasibility and quality issues are reported separately [5]. These issues included low confidence in the quality of data collected at one’s hub, concern that different hubs were not collecting data in the same way, and not yet having the ability to see trends, given only one data point. At least one respondent at each pilot hub reported that data issues were a barrier to the usefulness of the accrual metric for performance improvement at a hub and/or CTSA Consortium level.

I think as a metric across different sites [it] is only going to be useful if people are measuring it the same way.

I think more data points are always better. Well, the caveat is, if they are collected properly…it’s hard, because we just have one static point in time right now.

Data and sampling issues reduced the number of trials represented in the MAR. In 38% of hubs (3/8), the metric was calculated based on fewer than 20 eligible clinical trials. Given the small number of trials in the MAR relative to all clinical trials, several hubs questioned the representativeness of their metric value relative to their intended sampling frame, and therefore its usefulness for decisions about performance improvement.

Some respondents were concerned that efforts required to collect metric data would be so high there would be little or no resources available for performance improvement. Hubs also identified challenges to the use of surveys for collecting data about accrual, including nonresponse bias and/or low response rates, both of which decrease the generalizability of results and usefulness for performance improvement.

### Using the Median Value for Performance Improvement

Over three-quarters (78%) of respondents reported mixed views on the potential usefulness of the median value for the purpose of performance improvement (Table 1); that is, they thought using the median could be useful, but also described limitations or concerns. Nineteen percent of respondents described only limitations or concerns about using the median value and one respondent (3%) described only potential usefulness. Comparing respondent roles, a lower proportion of PIs had a mixed opinion.

**Table 1.** Opinion on the usefulness of the median value for performance improvement (*n* = 32)

		Opinion, *n* (%)		
	*n*	Useful	Mixed	Not useful
Team members	23	0	19 (83)	4 (17)
Principal investigators	9	1 (11)	6 (67)	2 (22)
**Total**	**32**	**1 (3)**	**25 (78)**	**6 (19)**

The most common barrier cited to using the median across trials for performance improvement was difficulty in interpreting the value. Many respondents indicated it is difficult to understand the meaning of a single number that represents a broad spectrum of trials. Several respondents found it difficult to use the median value to judge their hub’s performance without comparator data or a benchmark. A few respondents cautioned that even with comparison information, a high median value relative to other hubs does not necessarily mean there are no areas for improvement.

Some respondents noted that a median value may not change over time even when there are effective improvement efforts targeted toward a subset of trials. Another concern was that a CTSI, as one organization within a much larger institution, may engage with a small fraction of trials at the institution. As a result, some respondents concluded their CTSIs would have limited ability to influence change to improve the median ratio at their institution.

We are a relatively large academic medical center with a very large denominator of trials. So we simply don’t have the resources to help every – to say that we can move the median…would be a little naïve.

### Using the Accrual Ratio for Performance Improvement at an Individual Trial Level

Among 30 respondents who commented on the usefulness of the accrual ratio for performance improvement on the level of individual trials, only half thought it could be useful without noting limitations or concerns (Table 2). Forty percent described potential usefulness as well as limitations or concerns. The remaining 10% noted only concerns. Compared to team members, a higher proportion of PIs described limitations and concerns.

**Table 2.** Opinion on the usefulness of the accrual ratio for performance improvement at an individual trial level (*n* = 30)

		Opinion, *n* (%)		
	*N*	Useful	Mixed	Not useful
Team members	22	13 (59)	8 (36)	1 (5)
Principal investigators	8	2 (25)	4 (50)	2 (25)
**Total**	**30**	**15 (50)**	**12 (40)**	**3 (10)**

Most respondents were interested in using the accrual ratio to learn about recruitment at an individual trial level. Many also described ways to use individual trial data for performance improvement. The strategy most commonly described was to identify poorly accruing trials, and then provide support, if appropriate. An alternate option was to close studies with zero or low accrual ratios. One respondent thought individual trial data could be useful when allocating resources (e.g., study coordinators) within a department.

Many respondents had difficulty in understanding the accrual metric ratio and predicted that stakeholders, including PIs and leadership, would also have difficulty. Several respondents believed that training on the metric for various stakeholders would be required.

The idea of having four variables that are all part of a complex equation is not necessarily [an] easy thing to think about and to communicate to investigators when they want to know how we’re doing with trial accrual.

### Contextual Factors for Interpreting the Accrual Ratio

Contextual factors were also cited as important for interpreting accrual metric results. Many respondents noted that accrual is influenced by a variety of variables, such as study size, participant population, and type of treatment. A few respondents explained how recruitment for some studies varies by season, e.g., pediatric trial recruitment is typically higher during school summer vacation. For trials with a low accrual ratio, some respondents thought it would be necessary to follow-up with departments or individual study teams to understand study-specific contextual factors before determining whether a study could benefit from recruitment support.

In addition, several respondents noted that the metric does not account for the fact that accrual often occurs in a nonlinear fashion. For instance, recruitment could be very high during the initial stage of a study, but then drop off over time. A few respondents suggested incorporating considerations about where a study is in its timeline into metric interpretation and performance improvement. One hub only follows up on trials with no accrual if the trial has been open for at least 1 year; respondents explained that this approach gives studies “a chance to get going.”

### Using Subgroups to Facilitate Performance Improvement

Many respondents thought comparing accrual between subgroups (including departments or clinical trial offices, studies receiving CTSI services, or PIs with multiple trials) would be useful. Some of the suggested subgroups spanned across hubs, such as sites in a multi-site trial or studies on a certain disease across a specific geographic area. Potential ways to use subgroup-level data for performance improvement included:

- Allocate more resources to trial types that are less successful at recruitment.
- Request individuals from successfully accruing areas to mentor those in less successfully accruing areas.
- Compare accrual between trials that receive CTSA services with trials that do not receive these services to show the impact of a CTSA Program.
- For an accrual strategy implemented for a specific group of trials, compare accrual values between pre- and post-implementation to show the impact of the strategy.

### Aggregating Accrual Metric Results

Respondents were asked whether metric results from hubs should be aggregated across the CTSA Consortium, such as for comparison or benchmarking. The majority (63%) had a mixed opinion, indicating aggregating results could provide benefit, but also describing limitations and concerns (Table 3). Twenty-eight percent described only limitations or concerns; 9% described only benefits. Compared to team members, a higher proportion of PIs described limitations and concerns. There were no notable differences in the types of benefits, limitations, or concerns described by PIs compared with other team members.

**Table 3.** Opinion on whether accrual metric results should be aggregated across the CTSA Consortium (*n* = 32)

		Opinion, *n* (%)		
	*n*	In favour	Mixed	Opposed
Team members	23	2 (9)	16 (70)	5 (22)
Principal investigators	9	1 (11)	4 (44)	4 (44)
**Total**	**32**	**3 (9)**	**20 (63)**	**9 (28)**

The most common potential benefits cited of aggregating data were to use comparisons and benchmarks to help a hub assess its own accrual performance and to set hub-specific accrual goals. Many respondents, including those who saw the potential benefits in aggregating data, cautioned against aggregating data without considering context and data quality. According to respondents, study- and hub-level contextual factors should be incorporated to prevent inappropriate comparisons between dissimilar studies or hubs. Some thought it would be more appropriate to compare and benchmark within subgroups (e.g., by institution size, funder type, or content area). In addition, many respondents had low confidence in the quality of their hubs’ data and did not want to contribute their data to an aggregate dataset until the quality improved. Some of these respondents guessed that other hubs would also have low confidence in their data, and concluded that an aggregate dataset comprised of low-quality data would not be useful.

You may get into a scenario where you are comparing apples and oranges…so it will be really important to identify what the key demographics of an institution are in terms of their clinical trial portfolio, to be able to categorize people so that you can compare similar institutions.

Is it helpful to aggregate something where a lot of the contributors don’t have confidence in their data? You just end up getting an aggregate of something that you know you shouldn’t have confidence in, but then it takes on a life of its own because it’s written down on paper and people make decisions based on it; and it’s kind of risky.

Other potential benefits identified by hubs included the ability to leverage higher metric values to elicit research partnerships, determine the impact or value of multi-site initiatives or the CTSA Program, share learnings across hubs about accrual-related challenges and solutions, and develop accrual educational programs and resources.

In contrast, a few respondents worried that aggregated data could be used against their hub. They expressed concern that, if their hubs had lower metric values compared to others, NCATS could judge them negatively or industry could avoid partnering with them.

### Hub Suggestions to Improve Metric Usefulness

Several hubs thought that accrual should be tracked more frequently than annually to allow for more timely identification of and support to struggling trials.

The annual surveys aren’t going to help much because they are going to come late…If we don’t find out that somebody’s in deep trouble for a year…the ballgame may be already over, and the sponsor cancelled the contract and they’re off to another site.

According to respondents, electronic data systems, and tools (e.g., CTMS, electronic dashboard) would help make frequent data collection and reporting feasible. Additionally, some respondents believed it would be more useful to compute the metric with the most current data rather than retrospective data for the previous calendar year.

A few respondents suggested that an alternate accrual metric or approach would be more useful for performance improvement. According to one respondent, a target end date for recruitment is not usually specified for multicenter studies using a competitive enrollment design. For these types of trials, this respondent suggested replacing the denominator of the accrual ratio with the percentage of targeted patients accrued across all participating sites.

In general with a competitive enrollment multi-center study…the date that it’s scheduled to end is not important. What would be more important in terms of the comparator wouldn’t be ‘you’re 75% of the way through a time period’ as much as ‘75% of patients in the study nationwide have enrolled, and where are you in your recruitment compared to that?’

Respondents at another hub suggested analyzing accrual data on trials closed for recruitment to develop a metric that can reliably predict accrual at the end of a study’s recruitment period. These respondents believed that a predictive model would allow a hub to better identify trials that would benefit the most from recruitment support, i.e., those predicted to have poor final accrual.

## Discussion

We conducted a pilot test of the MAR metric with eight CTSA Consortium hubs to determine the usefulness of the metric result for conducting performance improvement for accrual at CTSA Consortium hubs and nationally. Interviews with the pilot hubs revealed common interests and concerns about using the accrual metric to address accrual performance. Hubs reported a great need to track and address accrual rates, and hoped that the metric could eventually be used to identify trials struggling with accrual for which a CTSA might intervene. However, issues of data quality (and representativeness of results, given small sample sizes), concerns about the metric’s design, and difficulty in interpreting the accrual metric value itself were seen as impediments to the metric’s ability to inform hub improvement activities. Considering contextual factors and comparisons with subgroups when interpreting metric results were thought to be important. Potential benefits to aggregating metric results across the CTSA Consortium included enabling comparisons or benchmarking, but most hubs acknowledged limitations or concerns with the aggregation of data from this metric.

We offer several recommendations to address the identified challenges.

**Continue to evaluate the usefulness of the MAR for performance improvement** and consider further revisions based on the assessments. Numerous issues, including data collection feasibility and data quality, limited hubs’ abilities to determine metric usefulness [5]. As data collection issues are addressed, hubs can learn and share opportunities to use metric results for improvement or identify needed metric revisions.

**Encourage more frequent assessment of accrual performance** to identify and provide services to trials in need of support to achieve their accrual targets within the planned timeframe. Using the metric only to evaluate accrual performance from a prior calendar year prevents hubs from taking action to intervene in trials not meeting accrual targets. Monthly or quarterly assessments would allow intervention closer to real time.

**Collect and report additional data to understand differences in accrual performance across trials and hubs**, including the total number of trials represented in the MAR, the percentage of total eligible trials included, and the mix of clinical trials by therapeutic area, study phase, type of study, and protocol complexity. These data are necessary to understand how representative the median is of the intended sample, and should inform decisions about aggregating data or comparing results across hubs.

**Consider additional accrual metrics to augment areas not addressed by the MAR**, such as time to the first participant accrued, the extent to which a trial is meeting race/ethnicity and gender accrual targets, and predictive metrics that identify trials likely to have low levels of accrual.

## Conclusion

In this pilot test of the Common Metric MAR, all participating CTSA Consortium hubs affirmed the importance of improving clinical trial accrual outcomes. However, significant challenges remain in ensuring that the metric can be sufficiently useful at the hub and CTSA Consortium levels to help realize the goal of collaborative process improvement. These challenges include:

(1) Interpretability: stakeholders may not readily understand the MAR or its key components, or believe that a single number can represent performance across a broad range of disparate trials;
(2) Actionability: it is unclear that the metric provides information that can lead to meaningful accrual improvement for trials, particularly among trials for which hubs have little ability to influence change;
(3) Comparability: heterogeneity between hubs in data sources, data collection strategies, and interpretations of metric definitions threatens the ability to aggregate or compare accrual performance across the CTSA Consortium; and
(4) Resource requirements: efforts to develop infrastructure to reliably collect data to construct the MAR, particularly for hubs without a CTMS, may leave little or no resources for accrual performance improvement.

Further metric testing could explore whether subgroups of trials (e.g., trial design, sponsorship, or hub involvement) exist for which the MAR is more useful or meaningful, potentially limiting those to which it is applied and concurrently reducing data collection burden.
